# Supplementary material for: GIPC3 couples to MYO6 and PDZ domain proteins, and shapes the hair cell apical region
Source: J Cell Sci. 2023 May 25;136(10):jcs261100. doi: 10.1242/jcs.261100 (PMC10234109; doi:10.1242/jcs.261100)
Supplement: Supplementary information [file joces-136-261100-s1.pdf]

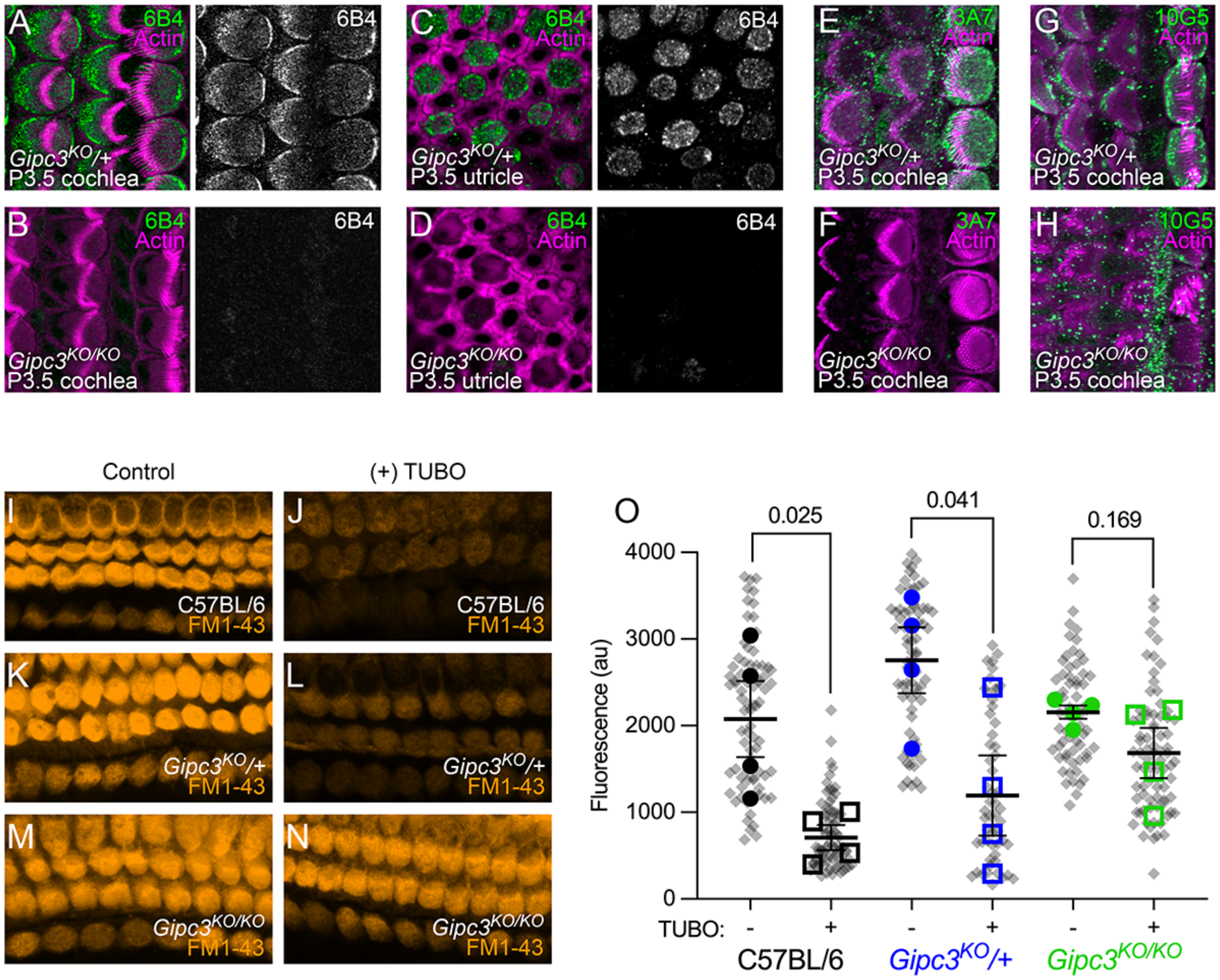


**Fig. S1. Anti-GIPC3 and *Gipc3*$^{KO}$ characterization. *A-H*,** Characterization of anti- GIPC3 monoclonal antibodies on mouse inner ear tissues. ***A-B***, 6B4 antibody on *Gipc3*$^{KO}$/+ and *Gipc3*$^{KO/KO}$ cochlea. ***C-D***, 6B4 antibody on *Gipc3*$^{KO}$/+ and *Gipc3*$^{KO/KO}$ utricle. ***E-F***, 3A7 antibody on *Gipc3*$^{KO}$/+ and *Gipc3*$^{KO/KO}$ cochlea. ***G-H***, 10G5 antibody on *Gipc3*$^{KO}$/+ and *Gipc3*$^{KO/KO}$ cochlea. ***I-N***, Images from FM1-43 labeling experiments. ***O***, FM1-43 signals determined for each hair cell (gray symbols). These cell measurements were averaged for each cochlea (11-29 cells per cochlea), and the averages were plotted with colored symbols (4 cochleas per condition) (Lord et al., 2020). Nested t-tests were used to compare the cochlea values for each genotype (Eisner, 2021); P values are indicated. Panel widths: A-H, 25 µm; I-N, 67 µm.

Journal of Cell Science • Supplementary information

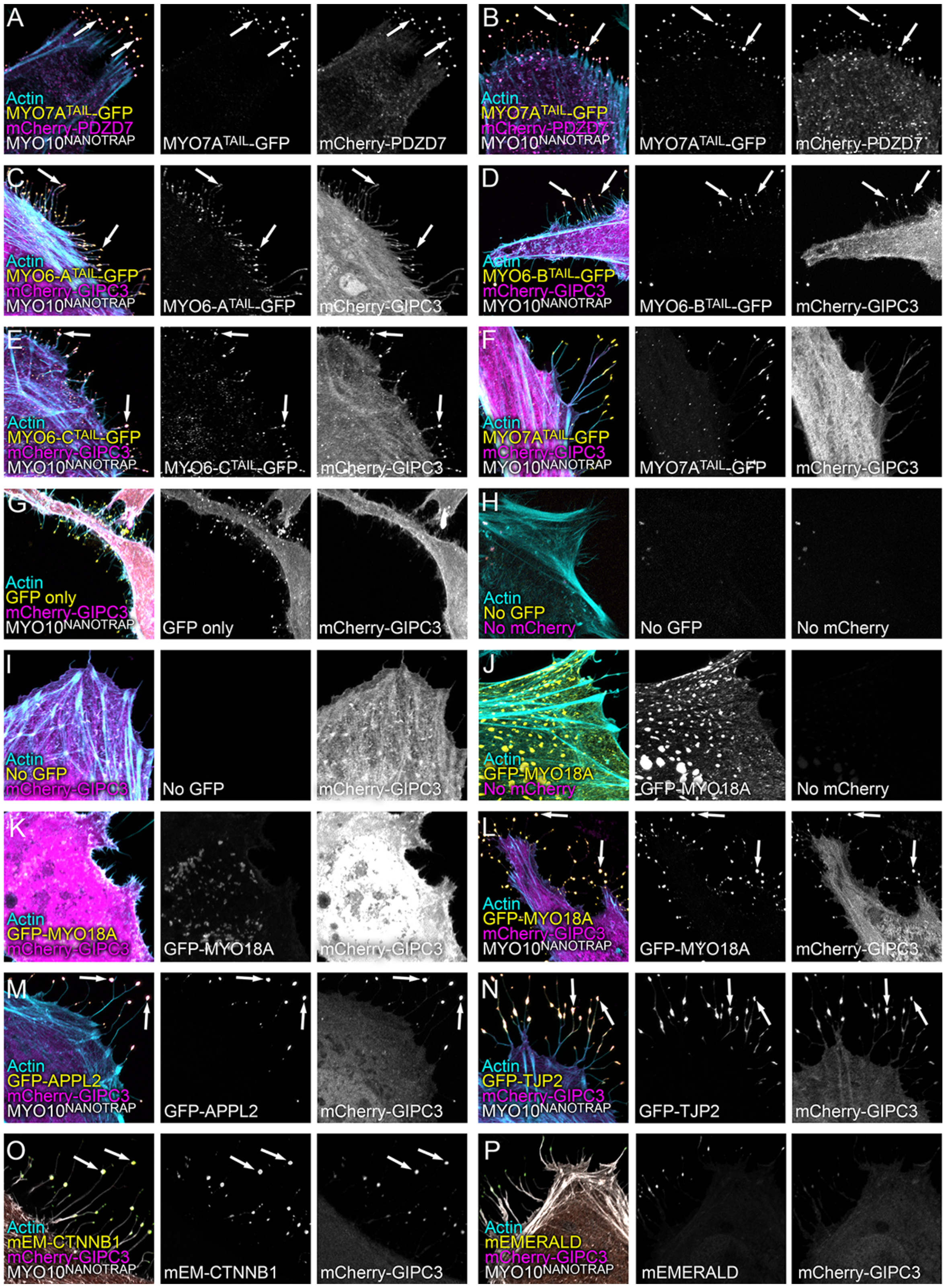


**Fig. S2. NanoSPD controls.** ***A-B***, Robust interaction (filopodial tip co-localization) between MYO7A$^{TAIL}$-GFP and mCherry-PDZD7 (two examples). ***C-E***, Robust interaction between A, B, or C splice forms of MYO6$^{TAIL}$-GFP and mCherry-GIPC3. ***F***, No interaction between MYO7A$^{TAIL}$-GFP and mCherry-GIPC3. ***G***, No interaction between GFP alone and mCherry-GIPC3. ***H***, No filopodia signal with negative control (no transfection). ***I,*** Cytoplasmic localization of mCherry-GIPC3 alone. ***J***, Cytoplasmic

Journal of Cell Science • Supplementary information

aggregates with GFP-MYO18A alone. ***K***, Cytoplasmic localization of GFP-MYO18A and mCherry-GIPC3 (no $MYO10^{NANOTRAP}$). Aggregates were present for GFP-MYO18A but the signal was reduced compared to the mCherry signal. ***L***, Robust interaction between GFP-MYO18A and mCherry-GIPC3. ***M***, Robust interaction between GFP-APPL2 and mCherry-GIPC3. ***N***, Robust interaction between GFP-TJP2 (ZO2) and mCherry-GIPC3. ***O***, Robust interaction between mEMERALD-CTNNB1 (mEM-CTNNB1) and mCherry-GIPC3. ***P***, Very weak interaction between mEMERALD and mCherry-GIPC3. For appropriate panels, arrows indicate filopodial tip co-localization. Panel full widths: 30 μm.

Journal of Cell Science • Supplementary information

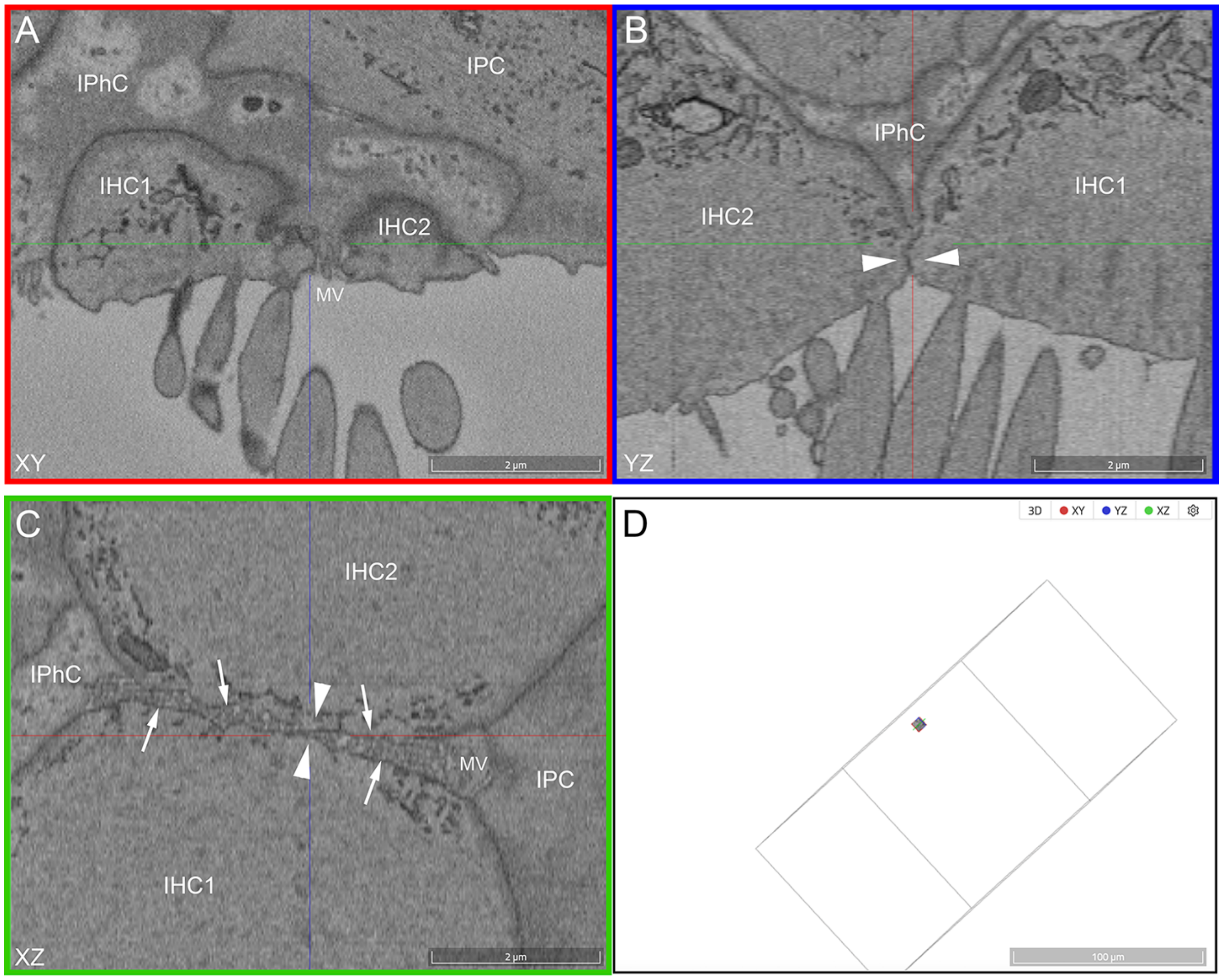


Data from: EM Reconstruction of Neural Circuitry in the Cochlea.
Y Hua, X Ding, H Wang, F Wang, Y Lu, J Neef, Y Gao, T Moser, H Wu.
Cell Reports. 5 January 2021. 10.1016/j.celrep.2020.108551

**Fig. S3. Relationship between apical domains of inner hair cells and inner phalangeal cells.** ***A***, X-Y plane. ***B***, Y-Z plane. ***C***, X-Z plane. ***D***, Location of the data presented within the entire dataset. Thin green, blue, and red lines show the transects for other image axes. Arrows indicate microvilli of inner phalangeal cells. Arrowheads indicate close IHC-IHC contact. The dataset used for this figure has been published (Hua et al., 2021) and is accessible at https://webknossos.org/datasets/b2275d664e4c2a96/HuaLab-CBA-mice-cochlea-mid-7w/#3954,4819,1586,0,37. Key: IHC, inner hair cell; IPC, inner pillar cell; IPhC, inner phalangeal cell; MV, microvilli.

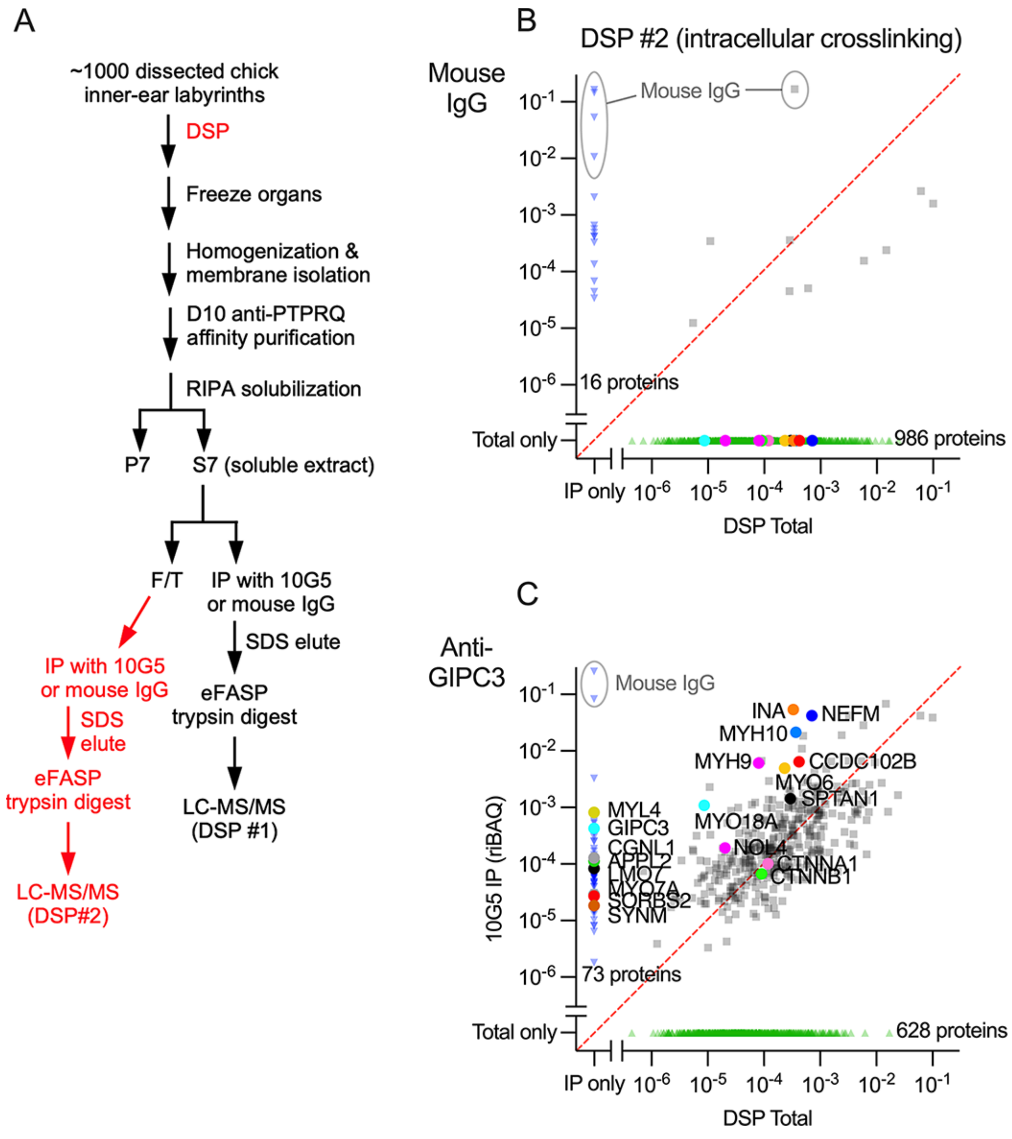


**Fig. S4. Anti-GIPC3 immunoaffinity chromatography from chick stereocilia extracts using DSP #2 extract.** ***A***, Flow chart for immunoaffinity chromatography, with new steps highlighted in red. The DSP #2 extract corresponds to the flow-through from the first DSP immunoaffinity chromatography step, rerun on either mouse IgG or 10G5. ***B-C***, Comparison of abundance (riBAQ) of proteins detected in total (starting DSP #2 S7 extract; plotted on x-axis) compared to the immunoprecipitates (y-axis) in mouse IgG control (B) and 10G5 anti-GIPC3 (C) experiments. Red dashed line is the unity line (equal riBAQ in total and IP). Key proteins are called out. Mouse IgG from immunoprecipitation is highlighted in gray.

Journal of Cell Science • Supplementary information

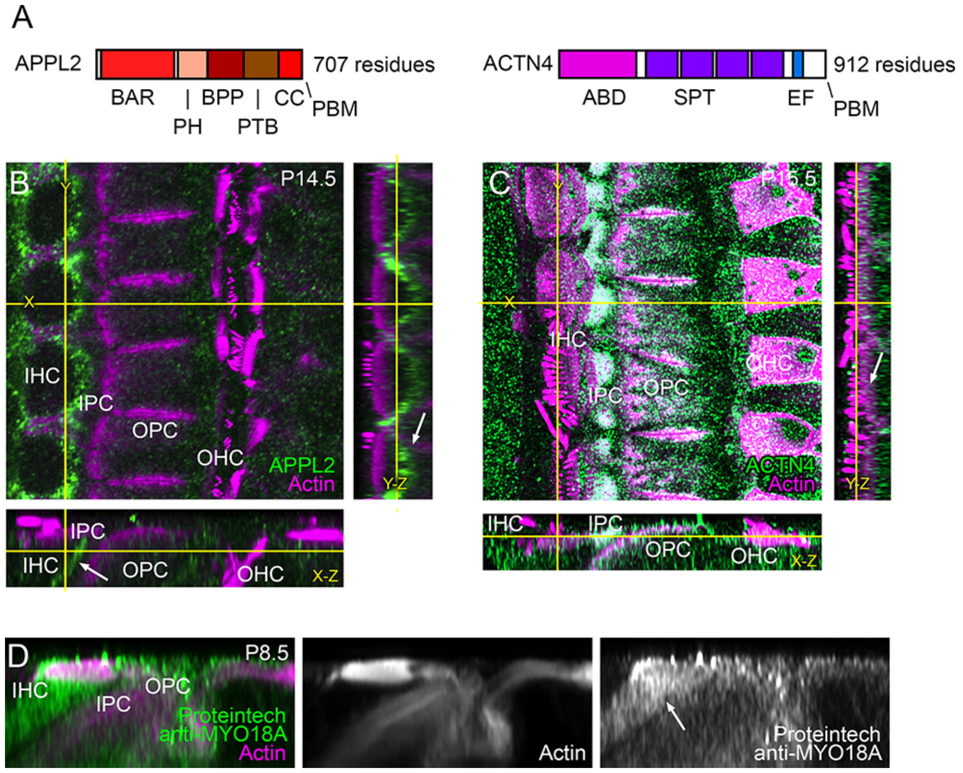


**Fig. S5. Localization of APPL2, ACTN4, and MYO18A in mouse cochlea.** ***A***, Domain structure of APPL2 and ACTN4. Key: BAR, Bin1/amphiphysin/rvs167; PH, pleckstrin homology; BPP, "region between PH and PTB domains;" PTB, phosphotyrosine binding; CC, coiled coil; PBD, PDZ- binding motif; ABD, actin-binding domain; SPT, spectrin domains; EF, EF hand. ***B***, Immunolocalization of APPL2 in P14.5 mouse cochlea; slices from a three-dimensional image stack. Transects for other image axes are shown in yellow; the X and Y transects in the main X-Y image show the locations for the Y-Z and X-Z images. Arrow indicates concentration of APPL2 immunoreactivity. Key: IHC, inner hair cell; IPC, inner pillar cell; OPC, outer pillar cell; OHC, outer hair cell. ***C***, Immunolocalization of ACTN4 in P15.5 mouse cochlea. Arrow indicates modest ACTN4 in IHC cuticular plates; the strongest labeling is in the head of the inner pillar cells and the sides of the outer pillar cells. immunoreactivity. ***D***, Labeling of IHC with Proteintech anti-MYO18A antibody, showing MYO18A distribution in the cuticular plate region. Key: IHC, inner hair cell; IPC, inner pillar cell; OPC, outer pillar cell; OHC, outer hair cell. Panel widths: X-Y, 37.5 μm (same scale applies to Y-Z and X-Z panels).

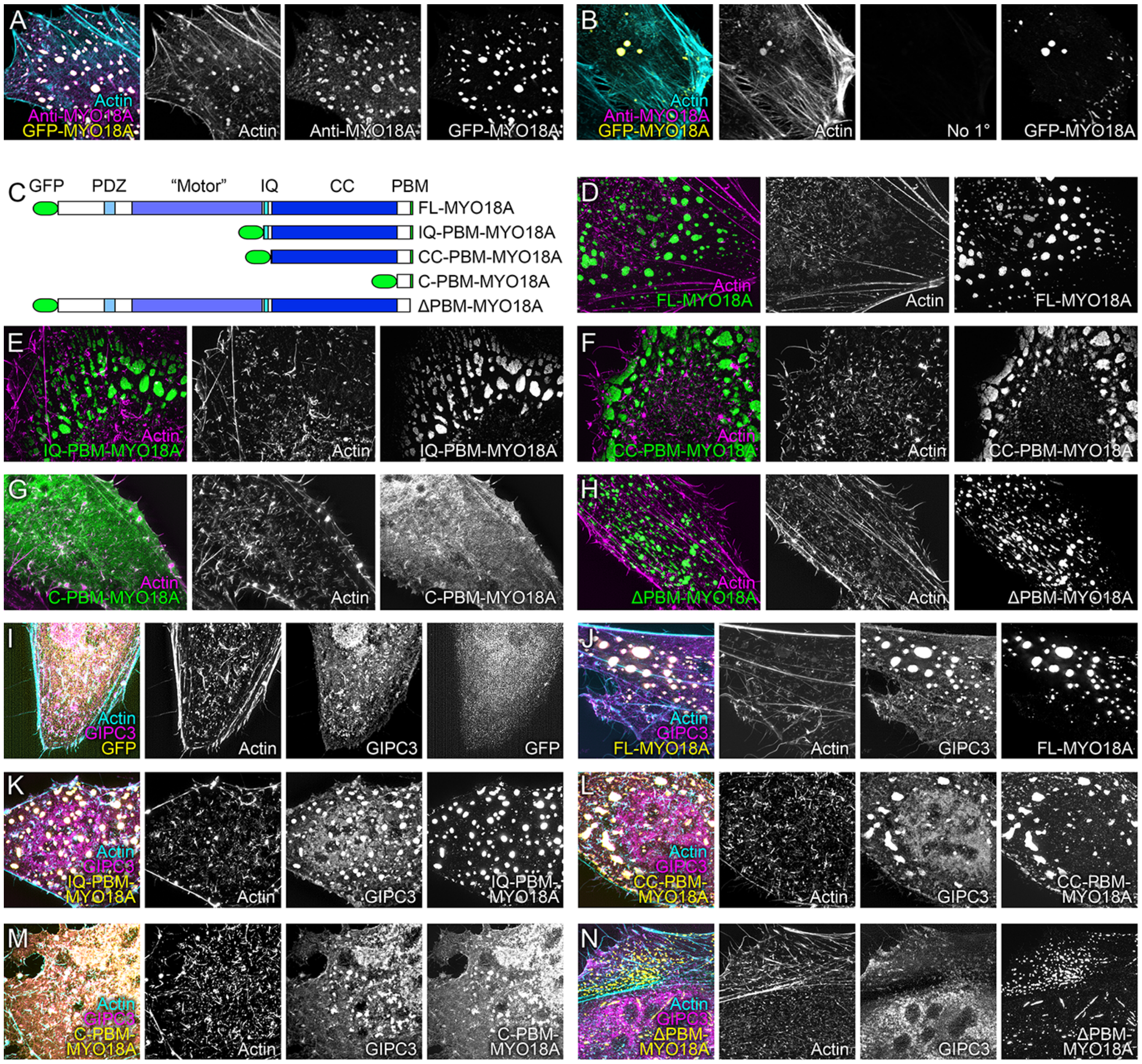


**Fig. S6. MYO18A forms aggregates and interacts with GIPC3 in the HeLa cell cytoplasm.** ***A-B***, Anti-MYO18A antibody specificity. ***A***, Anti-MYO18A detects the outer shell of GFP- MYO18A aggregates, but cannot penetrate into the core. ***B***, No signal in the absence of primary antibody. ***C***, Diagram of MYO18A expression constructs. The GFP fusion and MYO18A domains are indicated. Key: FL, full-length; IQ, calmodulin-binding IQ domain; CC, coiled-coil domain; C, C-terminal domain; PBM, PDZ-binding motif. ***D-H***, Expression of GFP-MYO18A constructs in HeLa cells. Co-labeled with phalloidin to show F-actin. ***I***, Co-expression of GFP and mCherry-GIPC3 in HeLa cells. ***J-N***, Co- expression of GFP-MYO18A constructs and mCherry-GIPC3 in HeLa cells. Panel full widths: A-B and I- N, 30 µm; D-H, 40 µm.

Journal of Cell Science • Supplementary information

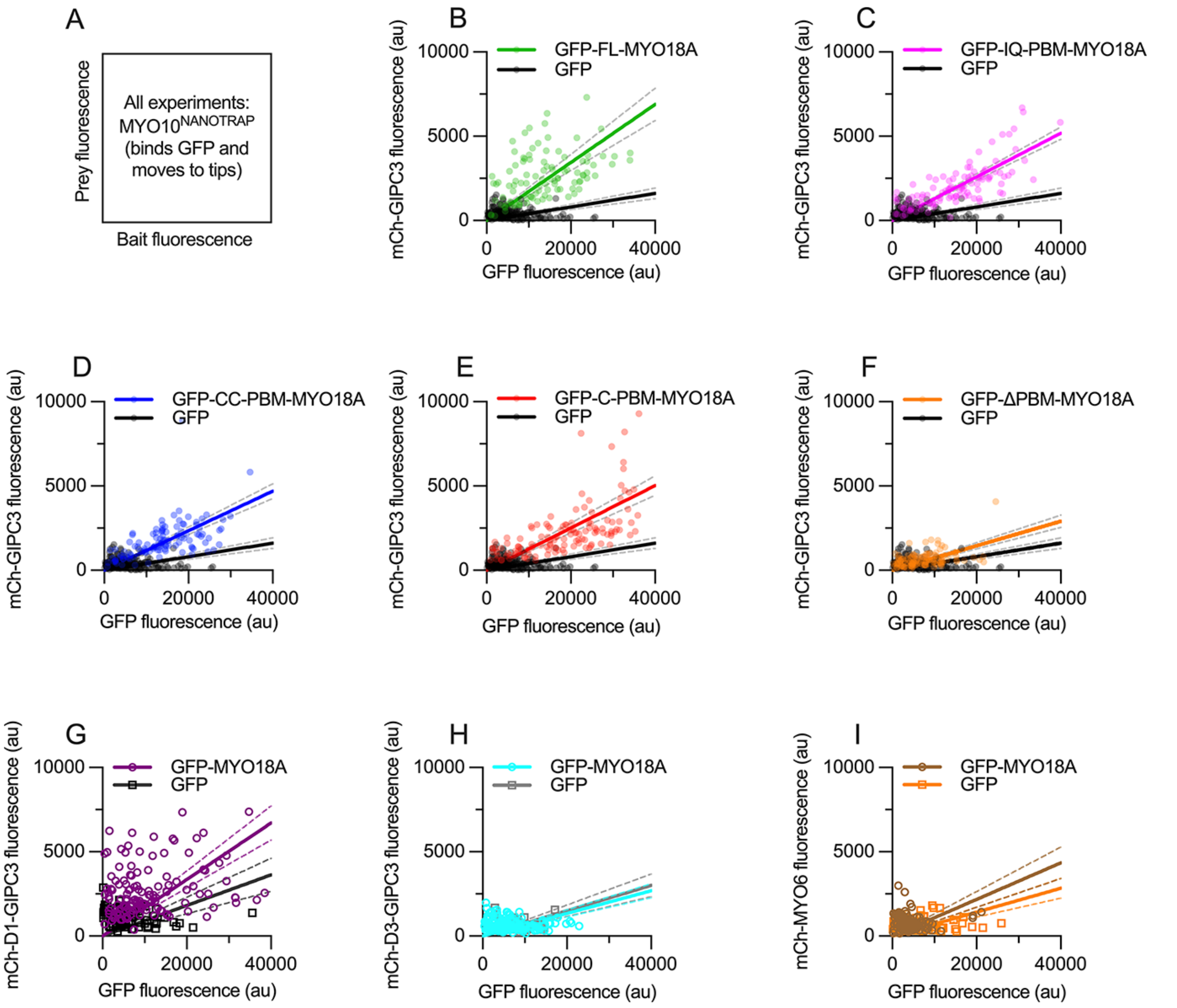


**Fig. S7. Intensity correlation analysis for MYO18A domain mapping by NanoSPD.** ***A***, Key for experiments and plots. ***B-F***, Intensity correlation analysis for interaction of mCh-GIPC3 with GFP-MYO18A constructs, using scatter plot of bait (x-axis) and prey (y-axis) fluorescence at individual filopodia tips (from three independent determinations). Dashed lines are 95% confidence intervals. ***G-H***, Intensity correlation analysis for interaction of GFP-MYO18A with mCh-GIPC3 constructs. ***I***, Intensity correlation analysis for interaction of GFP-MYO18A with mCh-MYO6 (negative control). Sample sizes were the same as in Fig. 8.

**Table S1. Plasmids used in nanoSPD experiments.**

| Plasmid name | Source | Vendor | Catalog # | Sequence info |
|---|---|---|---|---|
| pcDNA3.1-MYO10-HMM-Nanotrap | Commercial | Addgene | 87255 | https://www.addgene.org/87255/ |
| pEGFP-MYO7A-TAIL | Commercial | Addgene | 89585 | https://www.addgene.org/89585/ |
| EGFP-Myo6 Tail A | In-house | N/A | N/A | MYO6 tail domain (aa 901-1294): Splice variant A (delta aa 1147-1156) |
| EGFP-Myo6 Tail B | In-house | N/A | N/A | MYO6 tail domain (aa 901-1294): Splice variant B (delta aa 1036-1067) |
| EGFP-Myo6 Tail C | In-house | N/A | N/A | MYO6 tail domain (aa 901-1294): Splice variant C (delta aa 1036-1067 & aa 1147-1156) |
| mCherry-Gipc3 | Commercial | GeneCopoeia | Ex-Mn14117-M55 | Full length mouse GIPC3 |
| mCherry-Gipc3 Domain 3 (D3) | In-house; derived from GeneCopoeia Gipc3 plasmid | N/A | N/A | GIPC3 amino acids 221-291 |
| mCherry-Gipc3 Domain 1 (D1) | In-house; derived from GeneCopoeia Gipc3 plasmid | N/A | N/A | GIPC3 amino acids 92-297 |
| mEmerald-Beta-Catenin-20 | Commercial | Addgene | 54017 | Full length mouse CTNNB1 |
| mEmerald-control | In-house; derived from Addgene Beta-Catenin plasmid | N/A | N/A | mEMERALD tag |
| EGFP-Appl2 | Commercial | VectorBuilder | VB211013-1283jsd | Full length mouse APPL2 |
| EGFP-control | Commercial | Thermo Fisher Scientific | 12288015 | N-terminal GFP tag |
| mCherry-Myo6-A | In-house; derived from Myo6 Tail A | N/A | N/A | MYO6 tail domain (aa 901-1294): Splice variant A (delta aa 1147-1156) |
| EGFP-Myo18a | Commercial | VectorBuilder | VB211220-1232ekc | Full length MYO18A |
| EGFP-Myo18a-IQ tail | In-house; derived from VectorBuilder Myo18a plasmid | N/A | N/A | IQ domain of MYO18A |
| EGFP-Myo18a-CC tail | In-house; derived from VectorBuilder Myo18a plasmid | N/A | N/A | Coiled-coil domain of MYO18A |
| EGFP-Myo18a-c term | In-house; derived from VectorBuilder Myo18a plasmid | N/A | N/A | C-terminal end of MYO18A |
| EGFP-Myo18adelta4AA | In-house; derived from VectorBuilder Myo18a plasmid | N/A | N/A | MYO18A last 4 amino acids deleted |
| mCherry-PDZD7 | Gift from Mhamed Grati | N/A | N/A | Full length mouse PDZD7 |
| pEGFP-TJP2 | Commercial | Addgene | 27422 | Full length mouse TJP2 |

**Table S2. Genotype-phenotype comparison for G0 i-GONAD experiments.**

| | | Phenotype | | | | |
|---|---|---|---|---|---|---|
| Pup # | Genotype | Wild type | Ambig-uous | Mosaic | KO | Not exam-ined |
| 12 | Mosaic. Ex2, no sequence. Ex4, indel with no size estimate; large deletion, indel ≈8587bp. | | | | X | |
| 13 | Wild type | X | | | | |
| 14 | Wild type | X | | | | |
| 15 | Mosaic. Ex2 and Ex4, no PCR bands. Large deletion, indel with 8610bp deletion plus a 47bp insertion. | | | | X | |
| 16 | Wild type | | X | | | |
| 17 | Wild type | X | | | | |
| 18 | Wild type | | | | | X |
| 19 | Wild type | X | | | | |
| 20 | Wild type | | | | | X |
| 29 | Wild type | X | | | | |
| 30 | Wild type | | | | | X |
| 31 | Mosaic. Ex2 WT; Ex4, ≈5bp indel. | | | X | | |
| 32 | Wild type | | | | | X |
| 33 | Wild type | | | | | X |
| 34 | Wild type | | | | | X |
| 35 | Mosaic. No Ex2 band. Ex4 mosaic with 7bp indel. Mosaic large deletions (~8515bp deletion and 8573bp deletion + 59bp insertion). | | | X | | |
| 36 | Mosaic. Ex2, 378bp insertion. Ex4 PCR mosaic for WT and 199bp insert. | | | X | | |
| 37 | Wild type | X | | | | |
| 38 | Mosaic. Ex2 18bp deletion. Ex4 single T-to-A. Large deletion, an indel with 8560bp deletion and 40bp insertion. | | | | X | |
| 39 | Wild type | X | | | | |
| 40 | Mosaic. Ex2 17bp deletion. Ex4 single T insertion. Large deletion (8533bp). | | | | X | |
| 41 | Wild type | X | | | | |
| 42 | Mosaic. Ex2 50bp deletion. Ex4 single T-to-A, plus single T insertion. Large deletion (8560bp). | | | | X | |
| 43 | Mosaic. Ex2 60bp deletion. Ex4 single T insertion. Mosaic large deletions (~8560bp, ~8660bp, and ~8660bp deletion plus a 45bp insertion. | | | | X | |
| 45 | Wild type | | | | | X |
| 46 | Wild type | | | | | X |
| 47 | Wild type | | | | | X |
| 48 | Wild type | X | | | | |
| 49 | Wild type | | | | | X |
| 50 | Wild type | | | | | X |
| 51 | Wild type | | | | | X |
| 52 | Wild type | X | | | | |
| 53 | Mosaic. Ex2 indel (unknown size). Ex4 6bp deletion. Large deletion 8577bp. | | | | X | |
| 54 | Mosaic. Ex2 7bp indel . Ex4 mosaic for large deletion (8574bp). | | | | X | |

Summary: 10 mice with wild-type genotypes were examined, and all 10 had wild-type phenotypes; 11 mice with mosaic genotypes were examined, and all 10 had mosaic or knockout phenotypes.

Journal of Cell Science • Supplementary information

**Table S3. Proteins enriched in 10G5 anti-GIPC3 immunoprecipitates relative to chicken inner extract.** Only proteins detected in 2/2 technical replicates from one of the two experiments are displayed (35 total); proteins that were detected in the immunoprecipitates using control mouse IgG were excluded.

| Protein characteristics | | | DSP experiment #1 | | | | DTSSP experiment | | | |
|---|---|---|---|---|---|---|---|---|---|---|
| **Best protein identifier** | **Best protein description** | **Best protein symbol** | **De-tect-ed** | **riBAQ** | **Stoichi-ometry re GIPC3** | **IP/total** | **De-tect-ed** | **riBAQ** | **Stoichi-ometry re GIPC3** | **IP/total** |
| ENSGALP00000043100 | GIPC PDZ domain containing family, member 3 | GIPC3 | 2/2 | 4.14E-04 | 1.0 | IP only | 2/2 | 2.33E-03 | 1.0 | IP only |
| ENSGALP00000005475 | Oncomodulin (parvalbumin 3) | OCM | 2/2 | 2.22E-04 | 0.5 | IP only | 2/2 | 8.14E-04 | 0.3 | 1.7 |
| ENSGALP00000019801 | Septin 7 | SEPTIN7 | 2/2 | 1.71E-04 | 0.4 | IP only | 2/2 | 2.51E-04 | 0.1 | 8.5 |
| ENSGALP00000007822 | Coronin 1C | CORO1C | 2/2 | 1.49E-04 | 0.4 | IP only | 2/2 | 1.76E-05 | 0.0 | 2.2 |
| ENSGALP00000015531 | Dynein, cytoplasmic 1, intermediate chain 2 | DYNC1I2 | 2/2 | 1.36E-04 | 0.3 | IP only | 2/2 | 2.41E-05 | 0.0 | 2.1 |
| ENSGALP00000007644 | Myosin phosphatase Rho interacting protein | MPRIP | 2/2 | 1.14E-04 | 0.3 | IP only | 2/2 | 1.12E-05 | 0.0 | 2.6 |
| ENSGALP00000029968 | Ribosomal protein L3 | RPL3 | 2/2 | 1.05E-04 | 0.3 | IP only | 2/2 | 1.88E-04 | 0.1 | 0.7 |
| ENSGALP00000020645 | Adaptor protein, phosphotyrosine interaction, PH domain and leucine zipper containing 2 | APPL2 | 2/2 | 8.47E-05 | 0.2 | IP only | 2/2 | 3.16E-04 | 0.1 | 112.6 |
| ENSGALP00000006826 | Cingulin-like 1 | CGNL1 | 2/2 | 6.72E-05 | 0.2 | IP only | 2/2 | 3.60E-05 | 0.0 | IP only |
| ENSGALP00000001044 | Myosin VIIA | MYO7A | 2/2 | 6.36E-05 | 0.2 | IP only | 2/2 | 4.13E-04 | 0.2 | 15.5 |
| ENSGALP00000027304 | LIM domain 7 | LMO7 | 2/2 | 2.74E-05 | 0.1 | IP only | 2/2 | 6.19E-06 | 0.0 | 1.0 |
| ENSGALP00000033263 | Synemin, intermediate filament protein | SYNM | 2/2 | 2.63E-05 | 0.1 | IP only | 2/2 | 2.57E-05 | 0.0 | IP only |
| ENSGALP00000021860 | Sorbin and SH3 domain containing 2 | SORBS2 | 2/2 | 1.61E-05 | 0.0 | IP only | 2/2 | 7.49E-05 | 0.0 | 17.1 |
| ENSGALP00000023016 | LIM and calponin homology domains 1 | LIMCH1 | 2/2 | 1.16E-05 | 0.0 | IP only | 1/2 | 6.29E-06 | 0.0 | 2.6 |
| ENSGALP00000042590 | Keratin 18 | KRT18 | 2/2 | 1.80E-03 | 4.4 | 166.3 | 2/2 | 1.92E-03 | 0.8 | 7.1 |
| ENSGALP00000000419 _family | Family[1] (Internexin neuronal intermediate filament protein, alpha) | INA | 2/2 | 3.45E-02 | 83.2 | 104.0 | 2/2 | 1.14E-01 | 49.1 | 55.7 |
| ENSGALP00000038853 | Myosin XVIIIA | MYO18A | 2/2 | 7.76E-04 | 1.9 | 89.1 | 2/2 | 4.17E-04 | 0.2 | 39.4 |

Journal of Cell Science • Supplementary information

| | | | | | | | | | | |
|---|---|---|---|---|---|---|---|---|---|---|
| ENSGALP00000041153 | Keratin X | KRTX | 2/2 | 6.56E-03 | 15.8 | 72.4 | 2/2 | 1.07E-03 | 0.5 | 7.9 |
| ENSGALP00000020445 | Myosin, heavy chain 9, non-muscle | MYH9 | 2/2 | 5.11E-03 | 12.3 | 63.4 | 2/2 | 2.79E-03 | 1.2 | 18.9 |
| ENSGALP00000023352 | Myosin, heavy chain 10, non-muscle | MYH10 | 2/2 | 1.96E-02 | 47.3 | 52.6 | 2/2 | 1.01E-02 | 4.3 | 16.6 |
| ENSGALP00000030296 _family | Family[1] (Myosin, light chain 12A, regulatory, non-sarcomeric) | MYL12A; MYL12B; MYL9 | 2/2 | 5.30E-03 | 12.8 | 43.0 | 2/2 | 2.88E-03 | 1.2 | 6.3 |
| ENSGALP00000000422 | Neurofilament, medium polypeptide | NEFM | 2/2 | 2.81E-02 | 67.9 | 39.3 | 2/2 | 9.53E-02 | 41.0 | 41.4 |
| ENSGALP00000016648 | Keratin 78 | KRT78 | 2/2 | 1.74E-02 | 41.9 | 34.9 | 2/2 | 1.36E-02 | 5.8 | 5.7 |
| ENSGALP00000025573 | Myosin VI | MYO6 | 2/2 | 5.20E-03 | 12.5 | 22.3 | 2/2 | 8.29E-03 | 3.6 | 12.7 |
| ENSGALP00000024476 | Nucleolar protein 4 like | NOL4 | 2/2 | 4.37E-04 | 1.1 | 21.5 | 2/2 | 4.15E-04 | 0.2 | IP only |
| ENSGALP00000040890 | Sad1 and UNC84 domain containing 2 | SUN2 | 2/2 | 5.91E-04 | 1.4 | 17.0 | 2/2 | 2.78E-05 | 0.0 | 0.1 |
| ENSGALP00000014023 | General transcription factor IIH, polypeptide 4, 52kDa | GTF2H4 | 2/2 | 1.91E-03 | 4.6 | 15.8 | 2/2 | 1.62E-04 | 0.1 | 1.8 |
| ENSGALP00000023635 | Lamin B1 | LMNB1 | 2/2 | 6.45E-03 | 15.6 | 13.5 | 2/2 | 2.19E-04 | 0.1 | 0.7 |
| ENSGALP00000009801 | Lamin A/C | LMNA | 2/2 | 8.38E-04 | 2.0 | 12.0 | 2/2 | 3.37E-05 | 0.0 | 0.3 |
| ENSGALP00000023085 | Actinin, alpha 4 | ACTN4 | 2/2 | 6.49E-04 | 1.6 | 10.2 | 2/2 | 3.56E-04 | 0.2 | 2.0 |
| ENSGALP00000015409 | Actinin, alpha 1 | ACTN1 | 2/2 | 2.61E-04 | 0.6 | 10.0 | 2/2 | 1.24E-04 | 0.1 | 1.9 |
| ENSGALP00000030322 | Coiled-coil domain containing 102B | CCDC102B | 2/2 | 4.09E-03 | 9.9 | 9.7 | 2/2 | 3.81E-04 | 0.2 | IP only |
| ENSGALP00000022004 _family | Family[1] (Tropomyosin 3) | TPM3; TPM1; TPM2; TPM4 | 2/2 | 3.77E-04 | 0.9 | 8.8 | 2/2 | 4.13E-04 | 0.2 | 2.8 |
| ENSGALP00000036906 | Calcium/calmodulin-dependent protein kinase type II delta chain | CAMK2D | 2/2 | 1.53E-04 | 0.4 | 8.7 | 2/2 | 6.75E-05 | 0.0 | 1.1 |
| ENSGALP00000035478 | Filamin B, beta | FLNB | 2/2 | 4.48E-04 | 1.1 | 8.4 | 2/2 | 3.06E-04 | 0.1 | 3.8 |

[1] Protein families are defined as groups of proteins that share 20% or more of their identified peptides. The protein with the most evidence (unique peptides identified) is listed first.

**Table S4. Proteins interacting with GIPC1 and their C-terminal sequences.** The C-terminal ten amino acids of Ensembl splice forms of proteins identified as GIPC1 interactors in Katoh (2001) are listed. These sequences were used with Weblogo (https://weblogo.berkeley.edu/logo.cgi) to create the sequence logo in Fig 7.

| Species and Ensembl splice form | C-terminal |
|---|---|
| Hs PLXND1-201 | DNIYECYSEA |
| Hs LHCGR-204 | LLDKTRYTEC |
| Hs RGS19-202 | QGPSQSSSEA |
| Hs VANGL2-201 | VMRLQSETSV |
| Hs FZD9-201 | DPSLENPTHL |
| Hs FZD4-201 | KPGKGSETVV |
| Hs FZD2-201 | TNSRHGETTV |
| Hs APPL1-201 | EGGKKRESEA |
| Hs ITGA5-201 | QLKPPATSDA |
| Hs ITGA6-201 | SDKERLTSDA |
| Hs ITGA8-201 | QLTNDKTPEA |
| Hs NRP1-206 | LNTQSTYSEA |
| Hs SLC2A1-203 | FHPLGADSQV |
| Hs SEMA4C-201 | PDSNPEESSV |
| Hs SDC4-201 | KKAPTNEFYA |
| Hs IGF1R-217 | ALPLPQSSTC |
| Hs ADRB1-201 | RPGFASESKV |
| Hs CD93-201 | QYSPTPGTDC |
| Hs DRD2-202 | RKAFLKILHC |
| Hs ENG-202 | STPCSTSSMA |
| Hs LRP1-201 | PEDEIGDPLA |
| Hs LRP2-205 | ANLVKEDSEV |
| Hs NTRK1-206 | APPVYLDVLG |
| Hs TGFBR3-201 | STPCSSSSTA |
| Hs TPBG-201 | LTNLSSNSDV |
| Hs TYRP1-203 | KLQNPNQSVV |

## Supplemental References

**Eisner, D. A.** (2021). Pseudoreplication in physiology: More means less. *J Gen Physiol.* **153,** e202012826.

**Hua, Y., Ding, X., Wang, H., Wang, F., Lu, Y., Neef, J., Gao, Y., Moser, T. and Wu, H.** (2021). Electron microscopic reconstruction of neural circuitry in the cochlea. *Cell Rep.* **34,** 108551.

**Lord, S. J., Velle, K. B., Mullins, R. D. and Fritz-Laylin, L. K.** (2020). SuperPlots: Communicating reproducibility and variability in cell biology. *J Cell Biol.* **219,** e202001064.

Journal of Cell Science • Supplementary information
