# Supplementary material for: GIPC3 couples to MYO6 and PDZ domain proteins, and shapes the hair cell apical region
Source: J Cell Sci. 2023 May 25;136(10):jcs261100. doi: 10.1242/jcs.261100 (PMC10234109; doi:10.1242/jcs.261100)
Supplement: Supplementary file 1 [file joces-136-261100_review_history.pdf]

GIPC3 couples to MYO6 and PDZ domain proteins and shapes the hair cell apical region

Paroma Chatterjee, Clive P. Morgan, Jocelyn F. Krey, Connor Benson, Jennifer Goldsmith, Michael Bateschell, Anthony Ricci and Peter G. Barr-Gillespie

DOI: 10.1242/jcs.261100

Editor: Giampietro Schiavo

Review timeline

Original submission:	1 March 2023
Editorial decision:	11 April 2023
First revision received:	12 April 2023
Accepted:	13 April 2023

Original submission

First decision letter

MS ID#: JOCES/2023/261100

MS TITLE: GIPC3 couples to MYO6 and PDZ domain proteins and shapes the hair cell apical region

AUTHORS: Paroma Chatterjee, Clive P. Morgan, Jocelyn F. Krey, Connor Benson, Jennifer Goldsmith, Michael Bateschell, Anthony Ricci, and Peter G Barr-Gillespie
ARTICLE TYPE: Research Article

We have now reached a decision on the above manuscript.

To see the reviewers' reports and a copy of this decision letter, please go to: https://submit-jcs.biologists.org and click on the 'Manuscripts with Decisions' queue in the Author Area. (Corresponding author only has access to reviews.)

As you will see, the reviewers gave favourable reports, raising however some minorpoints that will require amendments to your manuscript. I hope that you will be able to carry these out because I would like to be able to accept your paper.

Please ensure that you clearly highlight all changes made in the revised manuscript. Please avoid using 'Tracked changes' in Word files as these are lost in PDF conversion.

I should be grateful if you would also provide a point-by-point response detailing how you have dealt with the points raised by the reviewers in the 'Response to Reviewers' box. Please attend to all of the reviewers' comments. If you do not agree with any of their criticisms or suggestions please explain clearly why this is so.

Reviewer 1

Advance summary and potential significance to field

The manuscript by Chatterjee et al identifies and characterizes inner ear hair cell molecular network involving GIPC3, a poorly studied protein with very important suspected function(s). The authors first found that GIPC3 is enriched at the apical pole of sensory hair cells since early

© 2023. Published by The Company of Biologists under the terms of the Creative Commons Attribution License (https://creativecommons.org/licenses/by/4.0/).

postnatal stages and that its absence in mouse model causes misshaping and distortion of inner and outer hair cell stereocilia bundle visible as early at P6-7 but preserving MET.
Authors also identified MYO6 as GIPC3 partner both colocalizing at hair cell apical junction at the pericuticular necklace area and that GIPC3 localization depends on MYO6, and not vice versa. Very interestingly, GIPC3-ko inner hair cells presented severe cuticular plate defects and altered apical junctions.
Besides, the authors identified GIPC3 interactome in hair cells several elements of which including MYO18A were localized in hair cells, therefore further demonstrating its effect on dimensions of cuticular plate and apical surfaces.
The scarcity of publications describing these types of molecular complexes and the dissection of their structural involvement clearly justifies the extreme technical and conceptual complexities of performing these studies. This study is extremely well-designed and beautifully executed, and convincingly demonstrates for the first time the role of GIPC3 and its molecular network in the shaping of hair cell cuticular plate and their apical junctions that are crucial in the stabilization, shaping, integrity and therefore excitability of mechanosensory stereocilia hair bundle.

Comments for the author

After a very thorough examination of the manuscript that is quite long and rich and utilized an impressive number of techniques and unique resources, I found the manuscript extremely well-written with beautiful figure panels that were nicely assembled into very informative sets of figures. I strongly recommend the immediate publication of this manuscript by JCS without any modifications or corrections. I congratulate in advance the authors for this impactful scientific achievement!

Reviewer 2

Advance summary and potential significance to field

Chatterjee et al.'s manuscript titled "GIPC3 couples to MYO6 and PDZ domain proteins and shapes the hair cell apical region" is a tour de force of cell biology, biochemistry, genetics, and functional analyses. Herein they show that GIPC3 localizes to the cuticular plate, a poorly understood organelle of hair cells, which deserves attention. The authors show that Gipc3KO/KO have defects in the cuticular plate and hair bundle morphology. They then use biochemistry/ cell biology to identify binding partners of GIPC3 and identify MYO6 and other proteins, such as MYO18A, that harbor PDZ binding motifs in their primary amino acid sequences. They produce an elegant model in Fig 8. The paper is important and sheds light on GIPC3's role in hair cells and perhaps beyond.

Comments for the author

Minor:
In Supplemental Figure S1, the panal corresponding to caption text is missing "P, IHC stereocilia count from SEM images for all three genotypes. Gipc3KO/KO bundles have reduced stereocilia numbers in all rows.
Sample sizes: Gipc3+/+ (2 cochleas; n=9), Gipc3KO/+ (6 cochleas; n=19), and Gipc3KO/KO (7 cochleas; n=25). Panel widths: A-H, 25 µm; I-N, 67 µm." Kindly modify figure accordingly.

Line 48: consider changing "a N-terminal" to "an N-terminal".

Line 89: consider defining the purpose of "data-dependent acquisition (DDA)".

Line 287: consider removing "the" before binary

© 2023. Published by The Company of Biologists under the terms of the Creative Commons Attribution License (https://creativecommons.org/licenses/by/4.0/).

First revision

Author response to reviewers' comments

Editor:
As you will see, the reviewers gave favourable reports, raising however some minorpoints that will require amendments to your manuscript. I hope that you will be able to carry these out because I would like to be able to accept your paper.

Please ensure that you clearly highlight all changes made in the revised manuscript. Please avoid using 'Tracked changes' in Word files as these are lost in PDF conversion.

> The last three minor points raised by Review #2 were corrected and highlighted with yellow in the revised text. The new version of the Supplemental Material has the text noted by Reviewer #2 removed.

I should be grateful if you would also provide a point-by-point response detailing how you have dealt with the points raised by the reviewers in the 'Response to Reviewers' box. Please attend to all of the reviewers' comments. If you do not agree with any of their criticisms or suggestions please explain clearly why this is so.

Reviewer #1:

Reviewer 1 Advance Summary and Potential Significance to Field: The manuscript by Chatterjee et al identifies and characterizes inner ear hair cell molecular network involving GIPC3, a poorly studied protein with very important suspected function(s). The authors first found that GIPC3 is enriched at the apical pole of sensory hair cells since early postnatal stages and that its absence in mouse model causes misshaping and distortion of inner and outer hair cell stereocilia bundle visible as early at P6-7 but preserving MET. Authors also identified MYO6 as GIPC3 partner both colocalizing at hair cell apical junction at the pericuticular necklace area and that GIPC3 localization depends on MYO6, and not vice versa. Very interestingly, GIPC3- ko inner hair cells presented severe cuticular plate defects and altered apical junctions. Besides, the authors identified GIPC3 interactome in hair cells several elements of which including MYO18A were localized in hair cells, therefore further demonstrating its effect on dimensions of cuticular plate and apical surfaces. The scarcity of publications describing these types of molecular complexes and the dissection of their structural involvement clearly justifies the extreme technical and conceptual complexities of performing these studies.
This study is extremely well-designed and beautifully executed, and convincingly demonstrates for the first time the role of GIPC3 and its molecular network in the shaping of hair cell cuticular plate and their apical junctions that are crucial in the stabilization, shaping, integrity and therefore excitability of mechanosensory stereocilia hair bundle.
Reviewer 1 Comments for the Author: After a very thorough examination of the manuscript that is quite long and rich and utilized an impressive number of techniques and unique resources, I found the manuscript extremely well-written with beautiful figure panels that were nicely assembled into very informative sets of figures. I strongly recommend the immediate publication of this manuscript by JCS without any modifications or corrections. I congratulate in advance the authors for this impactful scientific achievement!

> We thank the reviewer for this favorable assessment.

Reviewer #2:

Chatterjee et al.'s manuscript titled "GIPC3 couples to MYO6 and PDZ domain proteins and shapes the hair cell apical region" is a tour de force of cell biology, biochemistry, genetics, and functional analyses. Herein, they show that GIPC3 localizes to the cuticular plate, a poorly understood organelle of hair cells, which deserves attention. The authors show that Gipc3KO/KO have defects in the cuticular plate and hair bundle morphology. They then use biochemistry/ cell biology to identify binding partners of GIPC3 and identify MYO6 and other proteins, such as MYO18A, that harbor PDZ binding motifs in their primary amino acid sequences. They produce an

© 2023. Published by The Company of Biologists under the terms of the Creative Commons Attribution License (https://creativecommons.org/licenses/by/4.0/).

elegant model in Fig 8. The paper is important and sheds light on GIPC3's role in hair cells and perhaps beyond.

Thank you for this favorable review!

Minor:

In Supplemental Figure S1, the panel corresponding to caption text is missing "P, IHC stereocilia count from SEM images for all three genotypes. Gipc3KO/KO bundles have reduced stereocilia numbers in all rows.
Sample sizes: Gipc3+/+ (2 cochleas; n=9), Gipc3KO/+ (6 cochleas; n=19), and Gipc3KO/KO (7 cochleas; n=25). Panel widths: A-H, 25 µm; I-N, 67 µm." Kindly modify figure accordingly.

Good catch. We had previously removed those data from the figure and took out references to them from the text, but had forgotten to remove the supplemental figure legend text. The text is now deleted from the supplemental material file

Line 48: consider changing "a N-terminal" to "an N-terminal".

Corrected as suggested.

Line 89: consider defining the purpose of "data-dependent acquisition (DDA)".

This paragraph was rewritten to accommodate a definition of DDA.

Line 287: consider removing "the" before binary

Deleted.

Second decision letter

MS ID#: JOCES/2023/261100

MS TITLE: GIPC3 couples to MYO6 and PDZ domain proteins and shapes the hair cell apical region

AUTHORS: Paroma Chatterjee, Clive P. Morgan, Jocelyn F. Krey, Connor Benson, Jennifer Goldsmith, Michael Bateschell, Anthony Ricci, and Peter G Barr-Gillespie
ARTICLE TYPE: Research Article

I am happy to tell you that your manuscript has been accepted for publication in Journal of Cell Science, pending standard ethics checks.

© 2023. Published by The Company of Biologists under the terms of the Creative Commons Attribution License (https://creativecommons.org/licenses/by/4.0/).
